# Ethnography in Primary Health Care: Theoretical–Methodological Reflections on Pharmaceutical Services

**DOI:** 10.3390/pharmacy13050118

**Published:** 2025-08-28

**Authors:** Samara Jamile Mendes, Silvana Nair Leite, Livia Maria de Souza Gonçalves, Marília Berlofa Visacri, Silvia Storpirtis

**Affiliations:** 1 Department of Pharmacy, School of Pharmaceutical Sciences, University of São Paulo, São Paulo 05508-000, SP, Brazil; visacri.mb@usp.br; 2 Department of Pharmaceutical Sciences, Federal University of Santa Catarina, Florianopolis 88040-370, SC, Brazil; silvana.nair@hotmail.com (S.N.L.); liviamsouzagoncalves@gmail.com (L.M.d.S.G.); 3 Brazilian Society of Clinical Pharmacy, Brasília 70714-020, DF, Brazil; sstor2011@gmail.com

**Keywords:** pharmaceutical services, ethnography, primary health care

## Abstract

In pharmacy, theoretical and methodological approaches from anthropology and the social sciences have been increasingly used to understand the complexity of health–disease processes and their relationship with medicines and social practices. Ethnography offers a critical and in-depth lens for analyzing phenomena in Primary Health Care (PHC), bridging persistent gaps between theory and method in health research. This article presents the theoretical and methodological trajectory of an ethnographic study on pharmaceutical services in PHC, conducted through participant observation in three Units in São Paulo, totaling 166 h of fieldwork. Data were recorded in field diaries and analyzed using a thematic inductive approach, leading to the development of conceptual categories and an analytical framework. Reflections on the method enabled interpretive analyses based on assumptions that were confronted with national and international trends in pharmacy literature. Constructing the method in a non-isolated, context-sensitive way was essential to understanding how pharmacists actively shape their practices in PHC. The study reinforces the relevance of participant observation as both a methodological and interpretive strategy, revealing that pharmaceutical services are being constructed through culturally situated practices that respond to health needs with the pharmacist’s active involvement.

## 1. Introduction

In the field of pharmacy, a growing body of research has drawn on theoretical frameworks from anthropology and the social sciences to gain a deeper understanding of the complex nature of health–disease processes, their intersections with medicines and social experiences, and the potential contributions of pharmaceutical practice to improved health outcomes [1,2].

In this context, ethnography has made significant contributions to the health sciences, particularly in response to the need to explore social, symbolic, and cultural dimensions of health and care [3,4]. Rooted in anthropological traditions, ethnography is more than a research method; it is an epistemological stance that emphasizes immersion in the social world of the groups of people (or societies, cultures, or organizations) being studied. It involves the researcher becoming part of the social setting for an extended period, engaging in participant observation, and employing a range of qualitative techniques such as interviews, document analysis, and direct and unobtrusive observations. The goal is to generate a rich, naturalistic, and contextualized understanding of practices, meanings, values, and norms that shape everyday life within specific cultural environments. Ethnographic inquiry prioritizes the interpretation of the symbolic world of participants and seeks to uncover insights into how individuals make sense of their experiences. As such, it is not only a method for data collection but also a reflexive process shaped by the researcher’s interactions and position in the field [5]. Its object of study consists of the set of signifiers that are constructed, perceived, and interpreted by the researcher. For Geertz, an anthropologist devoted to exploring the interpretation of culture, being an ethnographer entails dealing with:

“(…) a multiplicity of complex conceptual structures, many of them superimposed upon or knotted into one another, which are at once strange, irregular, and explicit, and which he must somehow first grasp and then render. Doing ethnography is like trying to read (in the sense of ‘construct a reading of’) a manuscript, foreign, faded, full of ellipses, incoherencies, suspicious emendations, and tendentious commentaries, but written not in conventional graphs of sound but in transient examples of shaped behavior”.

[5] (p. 7)

Nonetheless, it is essential to produce studies that shed light on the methodological process of research, the construction of the field, and the practical execution of work that is based on human experience, that observes human beings, and is carried out by human beings. Choosing to conduct fieldwork is, for the researcher, a learning experience that should be shared, both as knowledge and lived experience—with all its challenges, discoveries, barriers, and achievements—within the exercise of sensitivity, proximity, and distance that characterizes the construction of the field [6].

Closer engagement with research participants makes ethnography a unique and highly complex methodology. The challenges involved in applying ethnography to health research stem largely from a positivist and reductionist perspective still present in the health sciences, in which qualitative research is often undervalued [3].

Pharmaceutical services in Primary Health Care (PHC) have evolved due to the integration of multidisciplinary health teams and pharmaceutical policies aimed at expanding access to medicines, particularly in local contexts [7]. Nevertheless, this is still an emerging field, most often explored through studies that examine the pharmacist’s role in health promotion, patients’ perceptions of PHC, and medicine-related needs [8,9,10].

Ethnography as a methodology provides a lens to guide researchers on this endeavor by offering richer insights into “real world practices” of professionals, while focusing on three key features of ethnographic analysis: thick descriptions, analytical generalizability and reflexivity. There has been little mention of ethnography or its potential as a specific research methodology [11]. However, qualitative research has been used as a modifier of health and pharmacy practice, but with little investment in theoretical foundations and analysis [12,13].

Given the persistent gap between theory and method in health research [4] and the potential of ethnography to offer a critical, reflective, and in-depth perspective on phenomena within PHC, this article aims to present the theoretical and methodological trajectory of an ethnographic study on pharmaceutical services in PHC. This work may serve as a reference for future ethnographic research and contribute to advancing methodological discussions in the field of health research.

## 2. Materials and Methods

This qualitative descriptive study presents the theoretical and methodological development of an ethnographic research project focused on pharmaceutical care in PHC, which was conducted and deeply immersed in by the first author (lead researcher) throughout the entire process [14].

The focus is not to report ethnographic research findings, but to provide a detailed account of the conceptual and procedural path that guided the development of the study—from its epistemological foundations to the choices made during fieldwork and analysis. The manuscript follows the Standards for Reporting Qualitative Research (SRQR) checklist [15], with emphasis on transparency and reflexivity in methodological reporting.

Ethnography remains an emerging approach in pharmaceutical research, despite its established relevance in the broader health and social sciences [11]. By systematizing and reflecting on the methodological trajectory of a completed ethnographic study, this article seeks to contribute to the field by sharing the rationale, challenges encountered, and solutions developed throughout the process.

A positionality statement is also included to clarify the researcher’s background and role: the ethnographer is a cisgender white woman who conducted participant observation during her Ph.D. at the University of São Paulo (USP); the researcher was unknown in the locations and disclosed her pharmacist background to participants at the case sites to foster transparency and trust, while engaging in continuous reflexivity regarding her own assumptions, perspectives, and subjectivities throughout the research process; her entry into the field of study was subject to institutional negotiation and received formal approval from the coordinators.

The study was approved by the Ethics Committee of the School of Pharmaceutical Sciences of the USP and the Municipal Health Secretariat of São Paulo/SP (SMS-SP). All participants signed an informed consent form.

The Results section presents the main elements of the theoretical and methodological pathway of the study, including details on data generation techniques, duration of fieldwork, and research settings. The Discussion provides a detailed exploration and reflection on the entire fieldwork process, situating it within the current scientific literature.

## 3. Results

The study was based on anthropological perspectives and guided by participant observation as a central methodological strategy.

In this study, field access was established through collaboration with various municipal health authorities in São Paulo, including the Coordination and Supervision departments of the SMS-SP, the management teams of PHC Units, and the Centers for Development, Education, and Research (CEDESP). These bodies assessed the feasibility of data collection, participant availability, and compliance with ethical standards.

Preparations for participant observation began with entering the field and conducting the study ethically from the outset. An essential step in this process was obtaining community permission to conduct the research, which involved introducing the study’s aims, namely, to document their daily activities, and presenting the informed consent form. Subsequently, the researcher worked to become unobtrusive and to build rapport with participants, fostering a sense of connection.

The gradual reduction of the community’s estrangement toward the researcher occurred over the span of a week. Nonetheless, interactions with health professionals and PHC Units were closely monitored, in accordance with ethical standards that require the research process to remain non-intrusive and not alter the phenomenon under investigation.

In this context, the observation can be classified as participant observation; however, in all three Basic Health Units, withdrawal from the field took place at a stage when interaction had become significantly intensified—to the extent that the researcher was occasionally mistaken for a member of the health care team.

Participant observation was conducted in three PHC Units in the city of São Paulo, Brazil. In Brazil, PHC is the central organizational strategy of the Unified Health System (SUS), a universal public health care system [16]. PHC services, including the pharmaceutical component, are decentralized and managed by municipal governments following guidelines established by the Ministry of Health [7].

The city of São Paulo is administratively divided into six health regions and has approximately 600 PHC Units, employing about 1700 pharmacists and 3000 pharmacy technicians [17]. Data collection took place in three PHC Units located in different regions of the city. Region 1 has an estimated population of 1,454,592, Region 2 approximately 2,553,802, and Region 3 around 2,380,783 inhabitants. Fieldwork was conducted between November 2017 and November 2018, totaling 166 h of observation, distributed across three cycles, one per PHC Unit. In this article, the field sites have been anonymized and are referred to as Case 1, Case 2, and Case 3, located in Regions 1, 2, and 3, respectively.

Case 1 was situated in a PHC Unit located in a residential neighborhood with limited commercial infrastructure, predominantly composed of houses and a few schools. The unit is located on a main avenue with relatively easy access to public transportation, al- though the nearest metro station is 11 km away. It is the only health care facility in the area and includes six Family Health Strategy (FHS) teams and one traditional PHC team. The pharmacy staff consists of one pharmacist (8 h/day), three pharmacy assistants (8 h/day), and two pharmacy technicians (6 h/day).

Case 2 was conducted in a PHC Unit located in a neighborhood characterized by numerous commercial establishments and five FHS teams. The pharmacy is staffed by one pharmacist (8 h/day) and two pharmacy technicians (6 h/day). 

Case 3 took place in a health unit that differs structurally from the previous ones and represents a specific model within São Paulo’s health care system: an Integrated PHC Unit–AMA Unit. This model integrates a PHC Unit with an Outpatient Medical Assistance Unit, which primarily addresses health demands not covered by standard PHC Units. It includes two FHS teams. The pharmacy team consists of one pharmacist (8 h/day) and four pharmacy technicians (6 h/day).

Observational data were recorded in three field notebooks, including detailed descriptions, informal conversations, contextual notes, and reflexive comments.

In this study, the researcher’s field notes captured the date, time, and location in each entry; used verbatim quotations whenever possible; assigned pseudonyms to protect participants’ confidentiality; described activities in chronological order; provided descriptions that did not influence the interpretation of events; incorporated relevant contextual information to situate the events; and clearly distinguished the researcher’s thoughts and assumptions from actual observations.

After extensive reading each field notebook, the notes were organized as follows: (1) pharmacist activities; (2) issues related to pharmacy technicians; (3) structure of the pharmacy and the PHC Unit; (4) methodological impressions; and (5) analytical notes (or instant analyses), resulting in 322 coded excerpts. The initial reading and organization of data were conducted using Atlas.ti ^®^ version 8 software.

The analytical process encompassed a progressive development and revision of assumptions, closely linked to the empirical material and the contextual realities of the observed services [14,18]. A thematic analysis was conducted through an inductive process. This involves a continuous, repetitive, and non-linear process of coding data into concepts, defining their properties, and grouping these concepts into categories.

The researchers’ insights into the phenomenon are presented in Table 1, which was instrumental in structuring ideas during fieldwork.

These insights into the phenomenon were continuously modified throughout the analytical process, as data emerging from the field revealed information about the activities carried out by pharmacists in PHC in the municipality of São Paulo, as well as pharmacists’ perceptions of their own professional practice. At the beginning of the fieldwork, it became evident that integration was occurring among all pharmaceutical services. However, it was necessary to further examine and understand how this integration took place, what factors interfered with it, and what interests were involved.

After completing the observation phase, the researcher stepped away from the field notebooks and turned her attention back to the specialized literature in the field of pharmacy, particularly in the areas of pharmaceutical services, clinical pharmacy, pharmaceutical care, and management [19,20,21,22,23]. This was essential for building new syntheses, as analyzing the phenomenon from different perspectives—and, especially, recognizing the observer’s positionality—is a critical step. The systematization and interpretation of empirical evidence were central to the ethnographic process, allowing the researcher to reconstruct key aspects of participants’ lived experiences.

No temporal distinction was made between data collection and analysis, as the latter was conducted concurrently with fieldwork. The empirical approach enabled iterative revisiting and refinement of the researcher’s perceptions. Table 2 presents the reformulated insights generated throughout the fieldwork, which constituted the foundation for the construction of the analytical grid.

Insights into the phenomenon are assessed through the sequential analysis of incidents, including potentially negative ones, allowing for the consolidation of research findings through inductive reasoning [18,24]. Thus, the analytical path is presented as shown in Table 3.

Coding becomes increasingly selective and theoretically integrated as analysis is ongoing through the review of all incidents. Some incidents correspond to the insights, while others relate to the reformulated insights. It is also important to highlight that when an incident was entirely new concerning the existing theory, there was no need to construct a negative case, since the incident itself served to refine the theory. An excerpt of the analytical framework developed during the analysis is shown as an example in Table 4.

It is important to highlight the following points during the analytical process:

1. Concepts are refined as they are shaped by the identification of field incidents and their contexts.
2. Field notebook entries represent the incidents—that is, complete actions or behaviors, whether individual or collective.
3. The concept reflects what the incident represents and requires interpretation rather than a mere explanation of the phenomenon itself.

For this reason, an analytical grid [25] was used as the theoretical/analytical framework derived from fieldwork and treated as an ethnological document.

## 4. Discussion

Qualitative research is inherently iterative and retroactive, as data collection, analysis, and the formulation of the research problem occur simultaneously, characterizing a model of continuous adaptation. Therefore, there is no precise point at which analysis begins, as it unfolds throughout the entire observation process [15].

This approach involves cultural immersion and the systematic documentation of social practices, allowing for the interpretation of the meanings attributed by the participants [5,26].

The trajectory of this participant observation began with the recognition of the need for critical reflection on the aspirations surrounding the development of pharmaceutical practice within PHC, particularly those related to pharmaceutical services and their advancement within the SUS.

One of the main contributions of Malinowski’s ethnographic approach lies in the centrality of participant observation as a tool for enabling ethnographic data collection. This involves the researcher embedding themselves in the community, gaining acceptance, and actively participating in events to understand the underlying logic that shapes group behavior. His methodological innovation of remaining in the field for extended periods allowed for in-depth analysis of the culture of the Trobriand islanders [26,27].

Entering and constructing the field involves choosing a site, obtaining authorization from those in charge, identifying key informants, and familiarizing oneself with the local setting or culture [15,26], as carried out with the municipal health authorities in São Paulo.

The establishment of rapport with the research field constitutes a fundamental aspect of the entry process. Only once this relationship has been developed—and the initial estrangement between the researcher and the field has subsided—can informed decisions be made regarding the focus of observation, culminating in a planned and ethically responsible exit from the field [28].

The researcher’s role in the field is a critical aspect to be examined, as it directly shapes the subjectivity inherent in participant observation. A central consideration is the way in which the observer engages with the research subjects. This study adopts an immersion-based ethnographic approach, in which observation is conducted through the researcher’s integration into the field, involving a process of socialization within the context under investigation [28].

The observation sample in this study was non-probabilistic or theoretical in nature and was intentionally constructed. This sampling strategy is commonly employed in ethnographic research to enable the identification of general aspects of the studied phenomenon, providing access to the situated and contextual knowledge of social life [29].

Field notebook entries include records of observations, informal conversations with participants, and documentation of activities, including those in which the researcher is unable to question participants directly, as well as daily field diary notes. The field or ethnographic diary, maintained systematically throughout the research process, is considered the ideal tool for data collection and analysis in this type of study [26].

A thematic analysis requires a detailed description of the studied situation, enabling the identification of meaningful properties of a given class of phenomena ([18], p. 349). The analytical process involves examining a series of cases, referred to as incidents, that may demand a gradual revision of the initial theory. Insights are constructed based on these incidents and refined throughout the research process until a consistent understanding of the observed phenomenon is achieved [28].

This approach aligns with ethnographic principles that emphasize understanding the totality of social life through immersion and reflexive analysis. Studying the whole does not mean studying everything, and totality can only be apprehended concretely through partial realizations, projected in human behavior [12].

Subsequently, an analytical grid was developed to present the theoretical framework resulting from the research [25].

Each analyzed incident represents a case, which may lead to a gradual revision of the overarching theory. In this way, negative cases are constructed with the purpose of refining the theory and progressively enhancing its capacity to explain the empirical data collected during the research [18].

The foundation of the analysis lies in the concepts formed through the coding of incidents, which are then grouped to construct conceptual categories. The concept does not designate the incident itself, but rather what it represents—its referent. A single observed incident may relate to multiple concepts. Concepts belonging to the same thematic universe are subsequently grouped into a conceptual category [18].

The theoretical and methodological contributions of anthropology to health research appear to be well established in the conceptual foundations of the ethnographic method and the potential for anthropological knowledge production. These elements serve as guiding principles for scientific rigor in health research, while also providing a framework to prevent risks of oversimplification [28].

Malinowski discusses the imponderables of real life in his work, referring to everyday phenomena that must be observed through the continuous engagement with the studied population (in his case, a tribe in New Guinea) [27].

The basis of participant observation, not only as a data collection technique but as a guiding strategy in qualitative studies, particularly those aiming to narrow the gap between discourse and the concrete practices of social actors, lies in a process of acculturation of the observer. This involves the assimilation of unconscious categories that structure the cultural universe under investigation [15,26].

Pharmaceutical services in Brazilian PHC have shown notable advancements in recent years. Scientific evidence has substantially contributed to this progress by highlighting improvements in the rational use of medicines by the population, as well as increased satisfaction among multidisciplinary teams regarding the role and performance of pharmacists.

Ethnography is a scientific approach that contributes to the production of evidence, particularly in the context of health practices targeting specific or vulnerable populations. In addition, it can inform health policies and decision-making processes, including those related to access to essential medicines management within health systems [30].

## 5. Conclusions

This study highlights the importance of using methodological strategies that involve participant observation in qualitative research, especially in the field of pharmacy, as they allow for reflections based on the perspective of the “Other”—the individual who experiences the issue being studied.

The development of pharmaceutical services in PHC can emerge from the codification of conceptual categories derived from thick description and culturally grounded understanding established in the real-world context.

The study’s insights into the phenomenon were inductively revised within the reality of PHC, ultimately forming a conceptual theoretical framework that allowed for a deeper understanding of the phenomenon of pharmaceutical services in PHC and the analysis of their interrelationships. This study aimed to present the trajectory and the theoretical-methodological frameworks underpinning this construction.

Based on these considerations, this work is expected to support researchers in the health field, particularly in the pharmacy field, in both the design (research planning) and practice (fieldwork, participant observation) of ethnographic research.

## Figures and Tables

**Table 1:** Initial insights into the phenomenon.

Initial Insights into the Phenomenon
Given that pharmaceutical services have been established as a major social policy that generates costs for the health system, and that their structuring, trained human resources, and access to medicines derive from this policy, it became necessary to reconsider the need to integrate pharmaceutical services into health care delivery, particularly within the logic of PHC.
One of the limiting factors in the management of pharmaceutical services is the predominance of a narrow, procedural view, which emphasizes their role as a medicine supplier and constrains their strategic function in promoting the rational use of medicines.
The medicine dispensing service should be integrated with other health care processes.
Clinical activities performed by pharmacists should be an integral part of pharmaceutical services.
Management should coordinate access and promote accessibility to both medicines and services.
Pharmaceutical services are mechanisms that contribute to achieving resolution in health care.
Pharmaceutical services involve the application of pharmacists’ knowledge and competencies for the benefit of others.

Legend: PHC = Primary Health Care.

**Table 2:** Reformulated insights emerging throughout the fieldwork process.

Reformulated Insights
Pharmaceutical services cannot be conceived in the same way across all contexts, as different needs and organizational models lead to context-specific constructions of pharmaceutical services.
The National Policy on Pharmaceutical Services in Brazil defines pharmaceutical services in a broad and integrated manner, prioritizing a set of actions aimed at ensuring access and the rational use of medicines: “it involves the research, development, and production of medicines and inputs, as well as their selection, planning, procurement, distribution, dispensing, quality assurance of products and services, monitoring, and evaluation of their use…”. Therefore, claiming that the activities of selection, planning, procurement, distribution, and dispensing are merely logistical and not aligned with a comprehensive health model reflects a disconnect from the broader construction of public health policies in Brazil.
Instead of using the terms “Clinical Pharmaceutical Services” or “Pharmaceutical Clinical Services,” the expression “Clinical services provided by pharmacists” is preferred.
Brazil presents a particular case regarding access to medicines through the SUS, and for this reason, people require pharmaceutical services related to management. Pharmacists understand that this is their responsibility within PHC and recognize it as fundamental to person- and community-centered care.
The pharmacist’s role has evolved in recent years; the focus of their services is no longer solely on medicines, but also on the individuals being served. In the case of PHC in Brazil, pharmacy practice is currently in a transitional phase toward the integration of clinical and managerial services, as it is expected that patients will have both proper access to medicines and appropriate, rational use of this resource.
Health needs are understood as a guiding axis for pharmacists’ work in PHC.
There is no ready-made model for pharmaceutical services, and fieldwork within PHC made this clear.
Pharmaceutical services have a broad conceptual scope and involve other professionals in both direct and indirect implementation.
It is normal for those experiencing the phenomenon under study to express discomfort, especially since universities have traditionally remained distant from the everyday realities of health services.

Legend: PHC = Primary Health Care; SUS = Brazilian Unified Health System.

**Table 3:** Example of an analytical framework for the phenomenon “Pharmaceutical Services in Primary Health Care” and its constituent elements.

Identification (Notebook–Incident)	Insights into the Phenomenon	Incident	Negative Case	Empirical Descriptions
1–47	Management coordinates access and promotes accessibility to medicines and services.	Dispensing of MMH.	Materials from the PAMG.	An activity that also falls under the pharmaceutical service needs. Some professionals must take responsibility; since the pharmacy has traditionally functioned as a storeroom, it has also had to assume this role.
2–14	Management coordinates access and promotes accessibility to medicines and services.	The pharmacist also stays in the PAMG room managing glycemic control; she helps with stock management of supplies, but her main focus is on the patients, as she can see their condition immediately by measuring glycemia on the spot.	An activity that also falls under the pharmaceutical service needs. Some professionals must take responsibility; al- though the pharmacy has traditionally functioned as a storeroom, in this case, F2 did more than just supply control—she also monitored patients.	A different service that takes up the pharmacist’s time, but can be used as potential for patient care.

Legend: PHC = Primary Health Care; F2 = Pharmacist from Case 2, MMH = Medical-Hospital Material, PAMG = Glycemic Self-Monitoring Program. The analysis of this section had no negative cases.

**Table 4:** Example of an analytical framework for the phenomenon “medicine dispensing” and its constituent elements.

ID	Insights into the Phenomenon	Reformulated Insights	Incident	Negative Case	Empirical Descriptions
2–50	Pharmaceutical services involve the application of pharmacists’ knowledge and competencies for the benefit of others.	*	One report mentioned the difficulty of staying in the dispensing area with the technicians and still carrying out other tasks, especially since the pharmacist does not carry out dispensing directly.	Pharmaceutical services require the application of pharmacist-specific knowledge and skills, but also draw on the expertise of other professionals, something highly feasible in PHC.	The pharmacist is not at the dispensing counter. How can this be structured?
2–124	*	Pharmaceutical services have a broad scope and involve other professionals in both direct and indirect execution.	Without F2, dispensing becomes operational, looking like just delivering products.	There is a risk of no clear responsibility for that service. If it is truly needed, both the community and the team recognize the importance of having a pharmacist.	Minimal pharmaceutical services occur in the pharmacy in the absence of the pharmacist.

Legend: PHC = Primary Health Care; F2 = Pharmacist from Case 2. * Cases in which there was no Insights into the phenomenon, but the incident led to the formation of a new concept.

## Data Availability

No new data were created or analyzed in this study. Data sharing is not applicable to this article.

## Abbreviations

The following abbreviations are used in this manuscript:

AMA	Outpatient Medical Assistance Unit
CNPq	National Council for Scientific and Technological Development
CEDESP	Centers for Development, Education, and Research
F2	Pharmacist from Case 2
FHS	Family Health Strategy
MMH	Medical-Hospital Material
PAMG	Glycemic Self-Monitoring Program
PHC	Primary Health Care
SMS	Municipal Health Secretariat
SRQR	Standards for Reporting Qualitative Research
SUS	Brazilian Unified Health System

## References

[1] Brown B., Undeberg M., Stewart A., McKeirnan K. (2025). Qualitative Analysis of Test-to-Treat Benefits and Barriers for Pharmacists in Rural Washington State. Pharmacy.

[2] Scott D., Hall A., Barenie R., Walker C., Khan M., Koltnow P., Callahan W.R., Cernasev A. (2025). “Put Me in, Coach”: A Discussion of Deprescribing Roles, Responsibilities, and Motivations Based on a Qualitative Study with Healthcare Professional Students. Pharmacy.

[3] de Novaes M.O., Moraes I.K.d.N., Rodrigues V.P., Vilela A.B.A., Casotti C.A. (2024). Ethnography and its contribution to research in health sciences. CLCS.

[4] Carnut L. (2019). Social research or qualitative research? A dis(de)cuss(construct)ion on the agenda in collective health. Saúde Debate.

[5] Geertz C. (1989). A Interpretação das Culturas.

[6] Leite S.N., Vasconcellos M.P.C. (2007). The construction of the research field: Reflecting on sociability among researcher and informers. Saúde E Sociedade.

[7] Mendes S.J., Farisco M., Leite S.N., Storpirtis S. (2022). A broad view of Pharmaceutical services in multidisciplinary teams of public Primary Healthcare Centers: A mixed methods study in a large city in Brazil. Prim. Health Care Res. Dev..

[8] Nazaryan L., Barseghyan A., Simonyan M. (2024). Evaluating Pharmaceutical Care Services in Promoting Public Health Outcomes within the Community. Indian. J. Public Health.

[9] Pereira N.C., Luiza V.L., Campos M.R., Chaves L.A. (2021). Implementation of pharmaceutical services in Brazilian primary health care: A cross-sectional study. BMC Fam. Pract..

[10] Al Zaidan M., Mohammed A.M., Mohamed Ibrahim M.I., Al Mahmoud M., Al Abdulla S., Al-Kuwari M.G. (2022). Pharmaceutical Care Service at Primary Health Care Centers: An Insight on Patient Satisfaction. Int. J. Clin. Pract..

[11] Lau S.R., Kaae S., Sporrong S.K. (2022). Ethnography and its potential for studying the social in social pharmacy: An example of autonomy and pharmaceuticals in eldercare. Res. Soc. Adm. Pharm..

[12] Lau S.R., Traulsen J.M. (2017). Are we ready to accept the challenge? Addressing the shortcomings of contemporary qualitative health research. Res. Soc. Adm. Pharm..

[13] Amin M.E.K., Nørgaard L.S., Cavaco A.M., Witry M.J., Hillman L., Cernasev A., Desselle S.P. (2020). Establishing trustworthiness and authenticity in qualitative pharmacy research. Res. Soc. Adm. Pharm..

[14] Pope C., Mays N. (2020). Qualitative Research in Health Care.

[15] O’Brien B.C., Harris I.B., Beckman T.J., Reed D.A., Cook D.A. (2014). Standards for Reporting Qualitative Research: A Synthesis of Recommendations. Acad. Med..

[16] Giovanella L., Franco C.M., de Almeida P.F. (2020). National Primary Health Care Policy: Where are we headed to?. Revista Ciência Saúde Coletiva.

[17] São Paulo (2024). Annual Management Report (RAG) of the SUS of the Municipality of São Paulo (MSP) for the Year 2023.

[18] Deslauriers J.P., Kérisit M., Poupart J., Deslauriers J.P., Groulx L.H., Laperrière A., Mayer R., Pires Á.P. (2014). O delineamento de pesquisa qualitativa. A Pesquisa Qualitativa. Enfoques Epistemológicos e Metodológicos.

[19] Soares L., Diehl E.E., Leite S.N., Farias M.R. (2013). A model for drug dispensing service based on the care process in the Brazilian health system. Braz. J. Pharm. Sci..

[20] Guimarães M.C.L., Santos S.M.C., Melo C., Filho A.S. (2004). Avaliação da capacidade de gestão de organizações sociais: Uma proposta metodológica em desenvolvimento. Cadernos de Saúde Pública.

[21] Pan American Health Organization (PAHO) (2011). Guidelines for the Development of Pharmaceutical Services in Primary Health Care.

[22] Brasil Ministério da Saúde (2014). Secretaria de Ciência; Tecnologia e Insumos Estratégicos; Departamento de Assistência Farmacêutica e Insumos Estratégicos. Serviços Farmacêuticos na Atenção Básica à Saúde.

[23] Marques D.C., Jeremias S.A., Storpirtis S., Mori A.L.P.M., Yochiy A., Ribeiro E., Porta V. (2008). Uma Carência no Sistema Único de Saúde (SUS): A Assistência Farmacêutica Íntegra.

[24] Desçauriers J.P., Poupart J., Deslauriers J.P., Groulx L.H., Laperrière A., Mayer R., Pires Á.P. (2014). A indução analítica. A Pesquisa Qualitativa. Enfoques Epistemológicos e Metodológicos.

[25] Guerra I.C. (2006). A diversidade de paradigmas de referência e os pressupostos das metodologias compreensivas. Pesquisa Qualitativa e Análise de Conteúdo. Sentidos e Formas de Uso.

[26] Kawulich B.B. (2005). Participant Observation as a Data Collection Method. Forum Qual. Sozialforschung/Forum Qual. Soc. Res..

[27] Malinowski B. (2018). Argonauts of the Western Pacific.

[28] Jaccoud M., Mayer R., Poupart J., Deslauriers J.P., Groulx L.H., Laperrière A., Mayer R., Pires Á.P. (2014). A observação direta e a pesquisa qualitativa. A Pesquisa Qualitativa. Enfoques Epistemológicos e Metodológicos.

[29] Nakamura E. (2011). The ethnographic method in health researches: An anthropological thinking. Saúde e Sociedade..

[30] Black G.B., van Os S., Machen S., Fulop N.J. (2021). Ethnographic research as an evolving method for supporting healthcare improvement skills: A scoping review. BMC Med. Res. Methodol..

